# Supplementary material for: Berberine Reveals Anticoccidial Activity by Influencing Immune Responses in Eimeria acervulina-Infected Chickens
Source: Biomolecules. 2025 Jul 10;15(7):985. doi: 10.3390/biom15070985 (PMC12292848; doi:10.3390/biom15070985)
Supplement: Supplementary file 1 [file biomolecules-15-00985-s001.zip › Supplementary Figure S1.pdf]

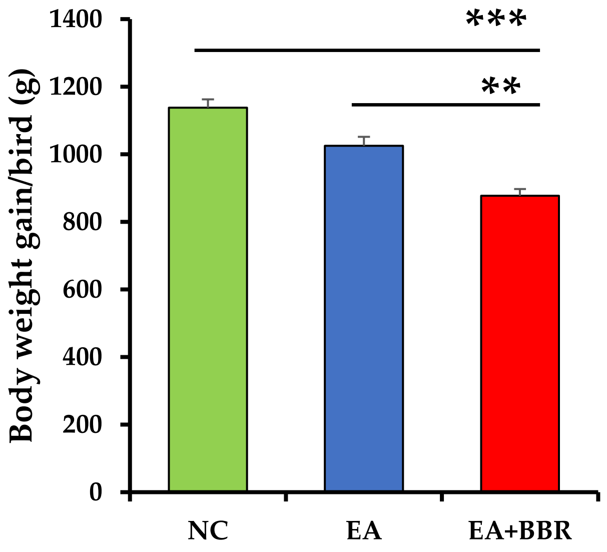

Supplementary Figure S1. Comparison of body weight gain in broilers after berberine treatment and *E. acervulina* infection. Chickens (n=10-20) were weighted at 9 days post-infection. NC, uninfected control; EA, *E. acervulina*; BBR, berberine. **$p < 0.01$ and ***$p < 0.001$.
